# Microscopic and Transcriptomic Comparison of Powdery Mildew Resistance in the Progenies of *Brassica carinata* × *B. napus*

**DOI:** 10.3390/ijms23179961

**Published:** 2022-09-01

**Authors:** Mingzheng Zhang, Qiong Gong, Xing Su, Yaohua Cheng, Haoxue Wu, Zhen Huang, Aixia Xu, Jungang Dong, Chengyu Yu

**Affiliations:** 1 College of Agronomy, Northwest A&F University, Taicheng Road 3, Yangling, Xianyang 712100, China; 2 College of Life Science, Nankai University, 94 Weijin Road, Nankai, Tianjin 300071, China

**Keywords:** rapeseed, cuticular wax, callose, Mildew Locus O, transcriptome, pre-penetration defense

## Abstract

Powdery mildew is a widespread disease in rapeseed due to a lack of resistant germplasm. We compared the foliar epidermal features and transcriptomic responses between the resistant (R) and susceptible (S) plants among the two parents and progenies of *Brassica carinata* × *B. napus*. The amount of cuticular wax and callose deposition on the R plants was much lower than that on the S plants; hence, these chemicals are not all essential to pre-penetration resistance, although the cuticular wax on the R plants had more needle-like crystals. A total of 1049 genes involved in various defense responses were expressed differentially among the R/S plants. The expression levels of two well-known susceptibility genes, *MLO6* and *MLO12*, were much lower in the R plant, indicating an important role in PM resistance. A set of genes related to wax biosynthesis (*KCS6*, *LACS2*, *CER* and *MAH1*), cell wall modification (*PMR5*, *PMEI9*, *RWA2*, *PDCB1* and *C/VIF2*), chloroplast function (*Chlorophyllase-1*, *OEP161*, *PSBO1*, *CP29B* and *CSP41b*), receptor kinase activity (*ERECTA*, *BAK1*, *BAM2*, *LYM1*, *LYM3*, *RLK902*, *RLP11*, *ERL1* and *ERL2*), *IPCS2*, *GF14 lambda*, *RPS4* and *RPS6* were highly expressed in the R plants. In the S plants, most highly expressed genes were involved in later defense responses, including *CERK1*, *LYK4*, *LIK1*, *NIMIN-1*, *CHITINASE 10*, *PECTINESTERASE*, *CYP81F2* and *RBOHF* and the genes involved in salicylic acid-dependent systemic acquired resistance and hypersensitive responses, indicating the occurrence of severe fungal infection. The results indicate that some uncertain pre-penetration defenses are pivotal for high resistance, while post-penetration defenses are more important for the S plant survival.

## 1. Introduction

Powdery mildew (PM) fungi are biotrophic pathogens that grow superficially on living host plants, coating the leaves, stems and fruits with spores and mycelia [1,2]. Cruciferous plant-specific PM disease caused by the pathogen *Erysiphe cruciferarum* is expanding year by year in various Brassica plants, especially rapeseed (*Brassica napus* L.), which is the second-largest oilseed crop in the world as well as a vegetable plant in East Asia. PM is the fourth most important disease causing heavy losses to rapeseed and is widely distributed over more than 25 countries around the world [1,2]. Growing disease-resistant varieties is an environmentally friendly and cost-effective strategy to control PM disease. However, there is no germplasm showing satisfactory PM resistance in rapeseed [2]; hence, resistance genes are desired by rapeseed breeders.

Plants have developed a well-orchestrated defense system against fungal attack. For example, cuticular wax acts as an important physical barrier to prevent fungi from entering the host cell [3], especially to PM, which grows superficially on plants [1,2]. Moreover, the host will show a series of immune responses including the accumulation of reactive oxygen species (ROS), H_2_O_2_, callose (β-1,3 glucan), pectin, cellulose and cuticular wax; the formation of papilla; the overexpression of resistance (R) genes and pathogenesis-related proteins; the phosphorylation of various kinases; the triggering of hypersensitive responses (HR); and the induction of systemic acquired resistance (SAR) [1]. The innate immune responses are controlled by pattern-triggered immunity (PTI) and effector-triggered immunity (ETI) [4]. Firstly, pattern recognition receptors (PRRs) on the cell surface of the host plant recognize pathogen-related molecular patterns (PAMPs) such as chitin and xylanase. For example, Arabidopsis LysM, containing receptor kinases (LYKs) including CERK1/LYK1, LYK4 and LYK5, is involved in the perception of chitin in the fungal cell wall [5,6]. Then, PRR activation triggers calcium-dependent protein kinase, a burst of ROS and phosphorylation of mitogen-activated protein kinase (MAPKs). The later plant responses include the expression of defense-related genes, biosynthesis of plant antibiotics and formation of papillae in the cell wall. The papilla is constructed by the callose layer, arabinoxylan and cellulose [7]. Overexpression of callose synthase-encoding genes *CalS12/PMR4* (*Powdery Mildew Resistant 4*) can enhance Arabidopsis resistance to PM [8,9]. Plant nucleotide-binding leucine-rich repeat receptors represent the majority of R proteins and they can recognize the effectors secreted by pathogens and then trigger ETI. ETI is often accompanied by HR, including oxidative burst, cell wall lignification and the induction of cell death (necrosis) in infected cells. Recent studies revealed some similar patterns of downstream responses and the interaction between the ETI and PTI systems [10].

The plant resistance to PM can be mediated by either R genes or the loss of function of susceptibility (S) genes, which are considered negative regulators of resistance, such as *Mildew Resistance Locus* (*MLO*) and *Enhanced Disease Resistance 1* (*EDR1*) [11,12,13]. On a barley *mlo* mutant, *Blumeria graminis* f. sp. *hordei* spores failed to penetrate host cells and did not form haustoria and secondary hyphae [11]. The features of pre-penetration resistance of the *mlo* mutant include the cell wall appositions, the formation of callose-rich papillae and mesophyll cell necrosis at penetration sites [11]. *PMR*s (*PM Resistance*) and *RPW8* (*Resistance to PM protein 8*) are important R genes in Arabidopsis. *PMR5* and *PMR6* encode putative pectin acetyltransferase and pectate-lyase-like protein, respectively, and they play roles in the defense against fungal pathogens [14]. RPW8.2 protein was induced by PM and it could stimulate the accumulation of hydrogen peroxide and callose at the host–pathogen interface [15].

In our previous study [2], an Ethiopian mustard (*Brassica carinata*) line, ‘Whiteflower’(denoted as WF), showed complete immunity to PM, and the PM resistance gene was transferred into the *B. napus* background of an elite cultivar, ‘Zhongshuang11’ (ZS11), through distant hybridization, backcrossing and resistance selection in the derived BC_1_F_3_ population, but the molecular mechanism of resistance remains unknown. In the present study, the characteristics of PM resistance in these plant accessions were studied through cytological observation of the foliar epidermal structure, detection of the transcriptional levels of several S/R genes and transcriptomic comparison among the S/R plants, to provide a theoretical and experimental basis to study the mechanism of PM resistance in Brassica plants.

## 2. Results

### 2.1. E. cruciferarum Pathogenesis on the Plant Accessions

**Figure 1:**
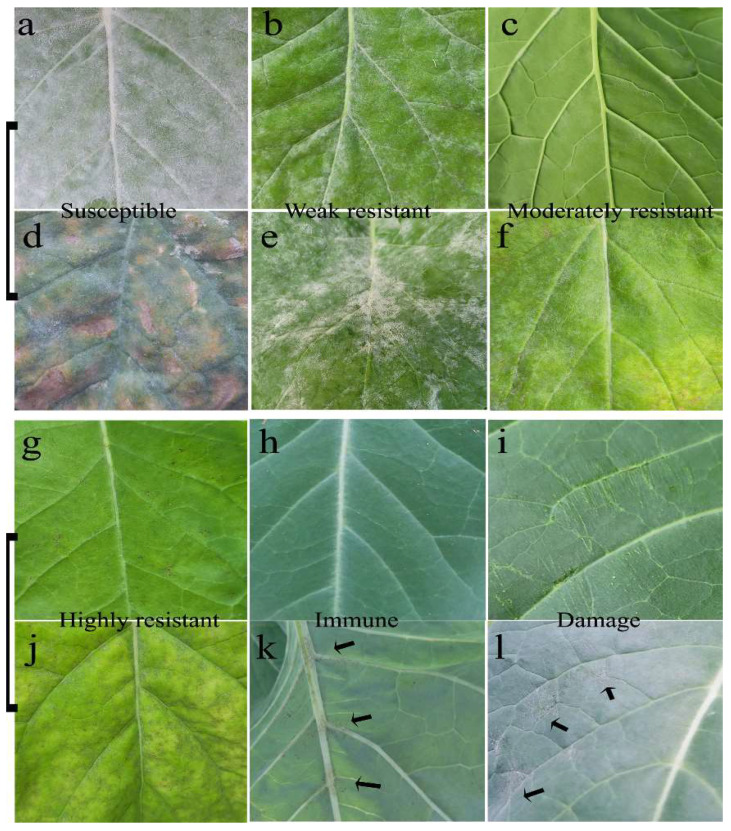
Phenotypic differences in the leaves of the different plant accessions after PM inoculation. More hyphae and mycelia were produced on the susceptible cultivar ‘ZS11’ (**a**) and the weakly resistant plant (**b**) than the moderately resistant plant in the BC_1_F_3_ population (**c**) and the highly resistant ‘BCWF’ (**g**). Necrotic lesions on the older leaves (**d**–**f**,**j**) of the above-mentioned plants. No hypha was found on the leaves of immune plant ‘WF’ (**h**), but we observed some brown dots on the older leaf of ‘WF’ (**k**, arrowheads). The spores could not penetrate the leaf of ‘WF’, where surface injury was caused by sandpaper friction (**i**), but mild infection (**l**, arrowheads) was observed on unhealthy plants with lateral root damage.

**Figure A1:**
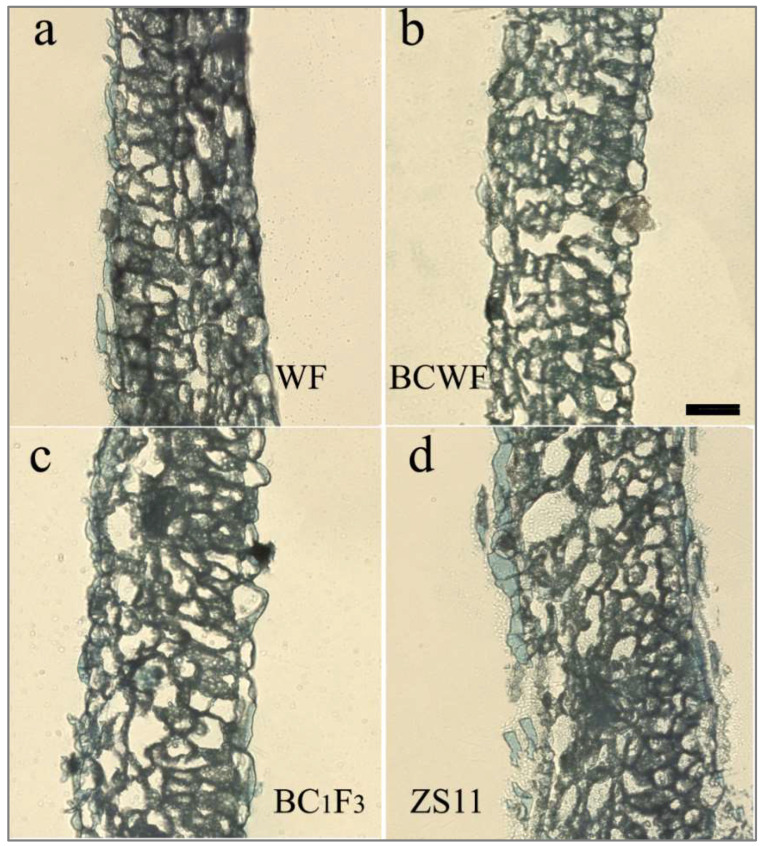
Transverse sections of the leaf samples of four plant accessions, ‘WF’ (**a**), ‘BCWF’ (**b**), ‘BC_1_F_3_’ (**c**) and ‘ZS11’ (**d**). The images have the same magnification and scale bar = 100 µm.

Compared to the modern rapeseed cultivar ‘ZS11’, Ethiopia mustard ‘WF’ and ‘BCWF’ (their F_1_ backcross to WF) had thinner leaves but a more compact foliar cell arrangement (Figure A1). The lower, older leaves of the susceptible plants were easily attacked by PM pathogen *E. cruciferarum*, but the undeveloped young leaves of all the tested plant accessions were never infected. The PM infection had obvious differences on the fully expanded leaves of different plant accessions after the inoculation (Figure 1). The susceptible cultivar ‘ZS11’ (Figure 1a) and the weakly resistant plant in the BC_1_F_3_ population (Figure 1b) showed more hyphae than the moderately resistant plant in the BC_1_F_3_ population (Figure 1c) and the highly resistant ‘BCWF’ (Figure 1g). Several weeks later, PM infection led to many necrotic lesions on the older leaves of these plants (Figure 1d–f,j). Hypha was seldom found on the leaves of ‘WF’ (Figure 1h) and only a few PM spores could form brown dots near the veins of the older leaf, especially on the abaxial epidermis (Figure 1k). It is believed that an existing aperture, such as a stoma or an injury on the host tissues, is able to be attacked by fungi [16], but we found that artificial injury by sandpaper friction did not help *E. cruciferarum* spores penetrate more easily into the epidermal cells of the ‘WF’ (Figure 1i). Exceptionally, the vulnerable plants in ‘WF’ due to root damage showed a mild PM infection (Figure 1l), indicating a possible association of water/mineral element deficits or photosynthesis inhibition with PM infection.

**Figure 2:**
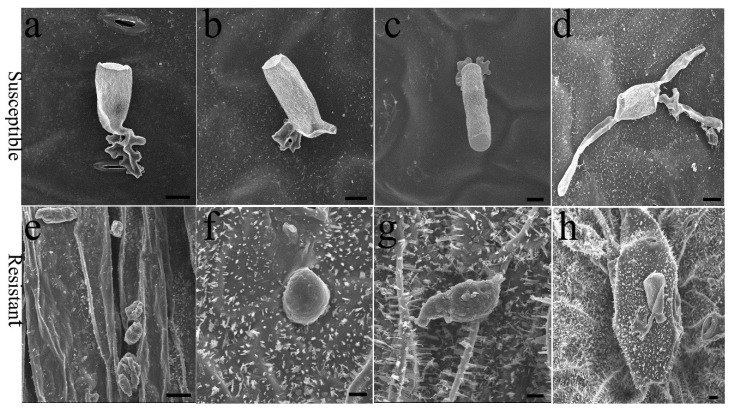
Scanning electron micrographs of *E. cruciferarum* developing on rapeseed leaves. Host cell penetration and formation of appressorium on the stomatal cell (**a**), pavement cell (**b**) or furrow between cells (**c**) of susceptible plant ‘ZS11’. Secondary hyphal growth after cell penetration (**d**). Dead spores on the foliar epidermis of the moderately resistant plant in the BC_1_F_3_ population (**e**), highly resistant ‘BCWF’ (**f**) and penetration-resistant ‘WF’ (**g**) after PM invasion. Scabs/nodules formed by the shrinkage and condensation of the tissue around the PM infection site (**h**). Scale bars represent 50 µm.

The *E. cruciferarum* spore has particular mechanisms for the attachment, identification and invasion of the host plant. When the spore alighted on the leaf, the early events of PM pathogenesis involved swelling and differentiating into a multi-lobed appressorium of the tip of the hyphal germ tube (Figure 2). On the susceptible plant, the events of appressorium formation and cell penetration could be found on the stomatal cells, pavement cells and furrows between adjacent cells (Figure 2a–c). Then, host cell penetration and the formation of haustorium enable the spore to absorb nutrients from the host plant. The second event in PM pathogenesis, the germination of secondary hyphae (Figure 2d), is a reliable indicator of successful cell penetration and haustorium differentiation, as a previous study suggested [17]. Compared to the ‘WF’ and ‘BCWF’, the susceptible ‘ZS11’ and the moderately resistant plants showed a very high fungal entry rate, with microscopically visible host cell penetration, the formation of secondary hyphae (Figure 2) as well as the establishment of PM colonies. Therefore, PM pathogenesis was not terminated early on the susceptible and moderately resistant accessions. On the highly resistant and immune plants, only very few spores could adhere to the abaxial surface due to drop into the furrows, but these spores died shortly without appressorium differentiation (Figure 2e–g). Occasionally, it was observed that on the abaxial epidermis of ‘WF’, some irregular nodules formed at the PM infection site (Figure 2h). To our knowledge, this unique morphological response to PM has not been reported in the previous literature. It would be interesting to dissect the wrinkled tissues, as well as cuticular wax, at the sites of spore attachment.

### 2.2. Difference in Foliar Cuticular Wax of the Plant Accessions

**Figure 3:**
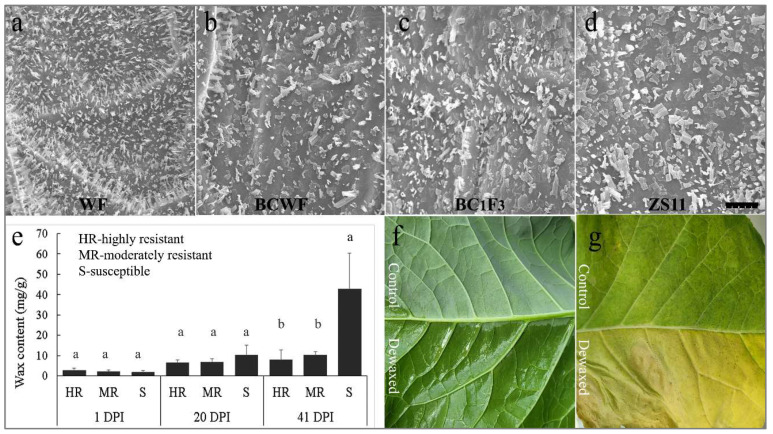
Comparison of the shape and content of the cuticular wax in different leaves and the effect of partially washing off the surface wax. The accessions in (**a**–**d**) are ‘WF’, ‘BCWF’, the moderately resistant plant in the BC_1_F_3_ population and ‘ZS11’, respectively. The cuticular wax of ‘BCWF’ and ‘WF’ is needle-like. The wax crystals of resistant plants in ‘WF and ‘BCWF’ are tubular-like or short and tubular, while that of ‘ZS11’ is mostly flake-like. The cuticular wax content in the leaves of plant accessions of different resistance levels (**e**). Different letters indicate significant differences (*p* < 0.05). Washing off surface wax from a half blade of ‘WF’ young leaf (**f**) leads to early senescence but not PM susceptibility (**g**).

The outer surface of the leaf is mainly composed of hydrophobic cuticular wax, while the inner layers are hydrophilic in composition. Observed under a scanning electron microscope, the surface wax on the sampled leaf was covered by flake-like waxes, decorated by some irregular granules of wax crystals (Figure 3). The crystals were mostly needle-like in the resistant plants of ‘BCWF’ and ‘WF’, but tubular-like in the moderately resistant plants in ‘W7-6’ and flake-like in the susceptible plant ‘ZS11’ (Figure 3a–d). This observation is in line with the results presented in Figure 2. Therefore, we assumed that the tubular- or needle-like crystal shape may be associated with PM resistance. The total leaf wax content increased when the leaf became older and PM infection became more serious at 20 and 41 days post-inoculation (DPI) (Figure 3e). In the early and middle stages of PM infection, the wax content on the resistant, moderately resistant and susceptible plants was at a similar level, but, in the later stage, the diseased leaves of susceptible plants had wax content significantly higher than that of resistant leaves (Figure 3e). However, it seemed that the penetration resistance in ‘WF’ does not require extra cuticular wax, because with the exception of early senescence, destroying and washing off the wax using laundry detergent did not lead to PM infection (Figure 3f,g) throughout the life span of the treated leaves, suggesting the presence of some defense mechanisms against PM infection besides cuticular waxes.

### 2.3. Callose Deposition on the E. cruciferarum Penetration Site

**Figure 4:**
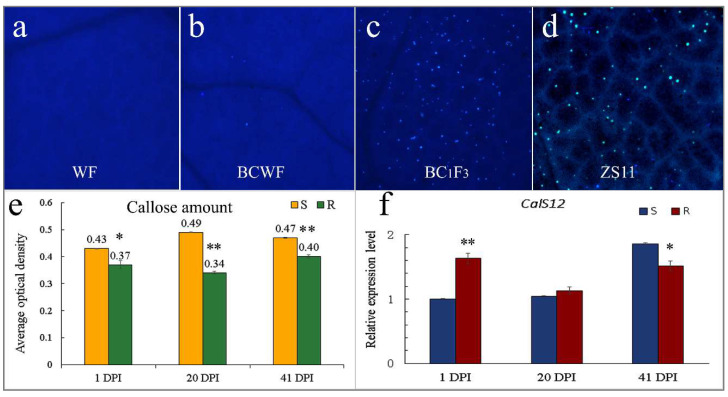
Infection-induced callose deposition and expression levels of two genes encoding callose synthases in the plants. Representative images for callose (particles stained with Aniline Blue) deposited on leaf surface of penetration-resistant ‘WF’ (**a**), highly resistant ‘BCWF’ (**b**), moderately resistant plant in the BC_1_F_3_ population (**c**) and the susceptible cultivar ‘ZS11’ (**d**) after inoculation with PM. (**e**) Callose deposition on the leaves of susceptible/resistant (S/R) plants in the BC_1_F_3_ population, quantified as fluorescence intensity per unit of the infiltrated leaf surface. Values represent means + SE of 10 different leaf samples. The expression levels of *CalS12* genes (**f**). Asterisks indicate statistically significant differences according to Student’s *t*-test (* and **, *p* < 0.5 and 0.01).

Callose deposition on the leaves of the inoculated plant accessions was observed by Aniline Blue staining (Figure 4). Callose deposition was seldom observed on the leaves of ‘WF’ and highly resistant accessions (Figure 4a); it was predominantly found on the moderately resistant and susceptible plants (Figure 4c,d). As calculated using the Image J software, we obtained an average optical density of 0.43, 0.49 and 0.47 for callose deposition on the S plants at 1, 21 and 42 DPI, being higher than 0.37, 0.34 and 0.40 on the R plants (Figure 4e). Callose was deposited at the PM penetration site, and each spot represented at least the successful cell penetration of PM. The intensity of callose fluorescence at the early stage of PM infection partially reflected the hypha amount at the early stage, correlating with tissue necrosis at the later stage. Thus, on the moderately resistant and susceptible plants, both callose deposition at the early stage and the above-mentioned necrosis at the later stage are important responses to PM penetration. On the contrary, callose was deposited to a much lesser extent on the immune accession ‘WF’ and resistant plants (Figure 4a,b), indicating a lower penetration rate than in the susceptible plants. Since callose synthase CalS12/PMR4 can strengthen the cell structure under stress conditions, expression analysis of the coding genes several days after PM inoculation was performed via qPCR. The expression level of BnCalS12 in the resistant accessions was kept at a relatively constant level, but in the susceptible plants the expression showed a gradual increase with the aggravation of PM infection (Figure 4f).

### 2.4. Transcriptomic Comparison of the S/R Plants in the Segregated Population

**Figure A2:**
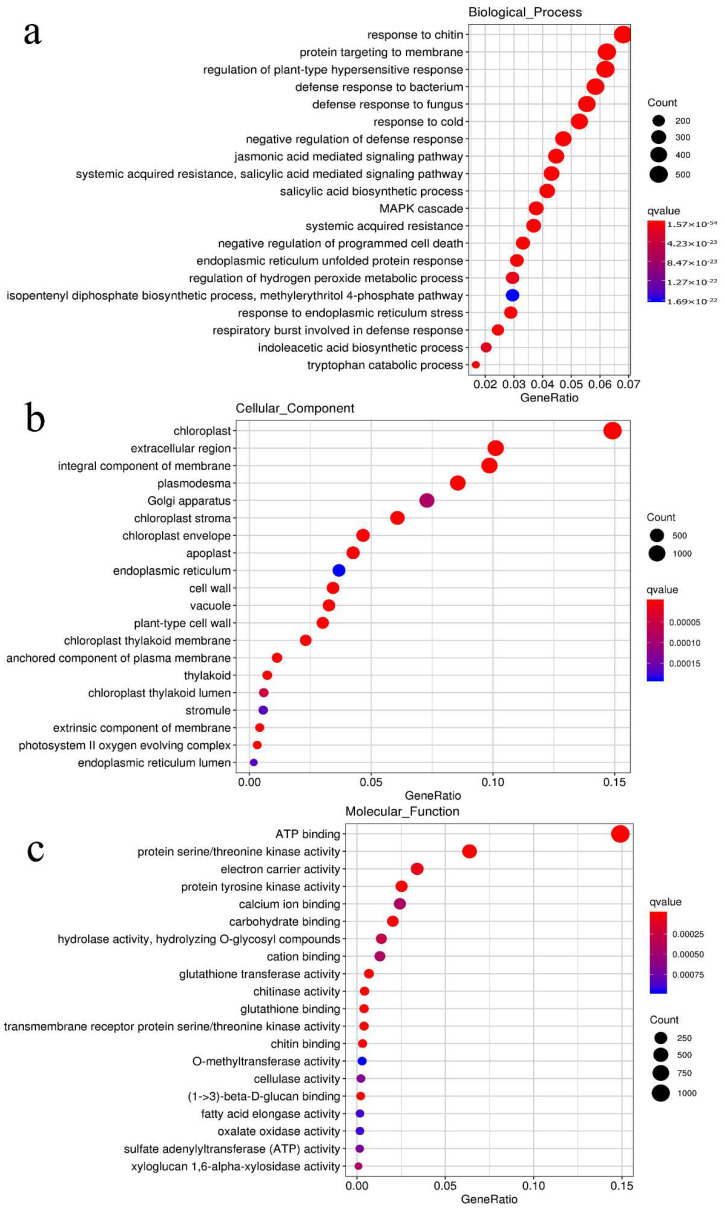
Dot plot of enrichment of GOs in biological processes (**a**), cellular components (**b**) and molecular function (**c**).

A total of 10,587 differentially expressed genes (DEGs) with Log_2_(foldchange) ≥ 1 and FDR < 0.01 were identified in the transcriptomic comparison of the R to S plants (NCBI accession GSE188377), of which 1049 DEGs are involved in disease resistance and defense responses, including 270 up-regulated and 779 down-regulated. The DEGs among the R/S groups were enriched in three main GO categories (Figure A2): biological processes (the top five GOs were response to chitin, targeting to membrane, regulation of hypersensitivity, response to fungus and SAR), molecular function (the three leading GOs were the activity of ATP-binding, kinases and electron carriers) and cellular component (the three leading GOs were chloroplasts, extracellular region and plasmodesma).

The highly expressed genes in the R plants (Dataset A1) mainly encode the proteins related to (1) the pathway of biosynthesis of the cuticular wax, such as LIPOXYGENASE 2 (LOX2), Β-KETOACYL-COA SYNTHASE KCS6/CER6, CER1(ECERIFERUM 1), LACS2, MAH1 (MID-CHAIN ALKANE HYDROXYLASE 1), GERMIN-LIKE PROTEIN GLP1 and GLP3; (2) the pathway of chloroplast structure and function, including CHLOROPHYLLASE-1/CLH1, OUTER ENVELOPE PORE PROTEIN 16-1/OEP161, OXYGEN-EVOLVING ENHANCER PROTEIN 1-1 PSBO1, CHLOROPLASTIC RNA-BINDING PROTEIN CP29B and CHLOROPLAST RNA BINDING PROTEIN CSP41B; and (3) the pathway of the construction and modification of the cell wall, including PMR5, CalS3, POLYGALACTURONASE INHIBITOR 1 (PGIP1), PECTIN METHYLESTERASE INHIBITOR 9 (PMEI9), REDUCED WALL ACETYLATION 2 (RWA2), PLASMODESMATA CALLOSE-BINDING PROTEIN 1 (PDCB1) and CELL WALL/VACUOLAR INHIBITOR OF FRUCTOSIDASE 2 (C/VIF2). Several genes encoding kinases such as RLK902, RKL1, ERECTA, BAK1, BAM2, LYM1 and LYM3, RLP11, ERL1 and ERL2, which recognize pathogens and trigger PTI immunity, were also expressed highly in the R plants. The genes encoding RPW8, 14-3-3-like protein GF14 Lambda (a positive regulator of RPW8-mediated PM resistance), RPS4 (resistant to P. syringae 4) and three copies of RPS6 homologs (BnaC04g31910D, BnaC02g32380D and BnaA06g35960D) were highly expressed in the R plants.

Meanwhile, another set of genes encoding CERK1 (Chitin Elicitor Receptor Kinase 1)/LYK1, LYK4, LysM RLK1-Interacting Kinase 1 (LIK1), NIMIN-1 (Nim1-Interacting 1), CHITINASE 10, PECTINESTERASE, ACCELERATED CELL DEATH 11 (ACD11), AUTOPHAGY-RELATED PROTEIN 8a, CYP81f2, 9s-LIPOXYGENASE (LOX1), Lipid transfer protein EARLI 1 (related to cutin biosynthesis) and RBOHF (generates ROS and regulates HR) were highly expressed in the S plants. Several genes encoding the most important proteins in the establishment of salicylic acid (SA)-dependent systemic acquired resistance (SAR), such as fatty acid dioxygenase DOX1, ICS1 (Isochorismate synthase 1/Enhanced disease susceptibility 16), NUDT7, SARD1 (SAR DEFICIENT 1), EDR2 (Enhanced Disease Resistance 2, a negative regulator of SA-based defenses and cell death during PM infections), EDR2-like, EDR4 (a negative role in SA-mediated resistance to PM), PDLP5 (negative regulator of plasmodesmata permeability) and WRKY70 (positively regulates the SA-mediated signal) were activated in the S plants. A set of genes encoding TIR-NB-LRR receptor-like protein RML1B, RPP1 (RECOGNITION OF PERONOSPORA PARASITICA), RPP4, RPS5, RPS6, DSC1, DSC2, PR-1 (a marker of plant immune signaling), HR3 (homolog of RPW8 3), RLP23, bZIP22/TGA3, NPR3, NPR4, ligase GH3.12 (required for HR), detoxification protein EDS5, Defensin-like protein 2, Defensin-like protein 4, WRKY50, WRKY53 and important kinases (MEKK1, MKK1, MKK2, MKK4, MPK4, RLK6/CRK5, CRK45, APK1, BRl3, WAK1 and WAKL10) were the most activated genes in the S plants (Dataset A1).

### 2.5. Protein Interaction Network Estimated by the Differentially Expressed Genes

**Figure 5:**
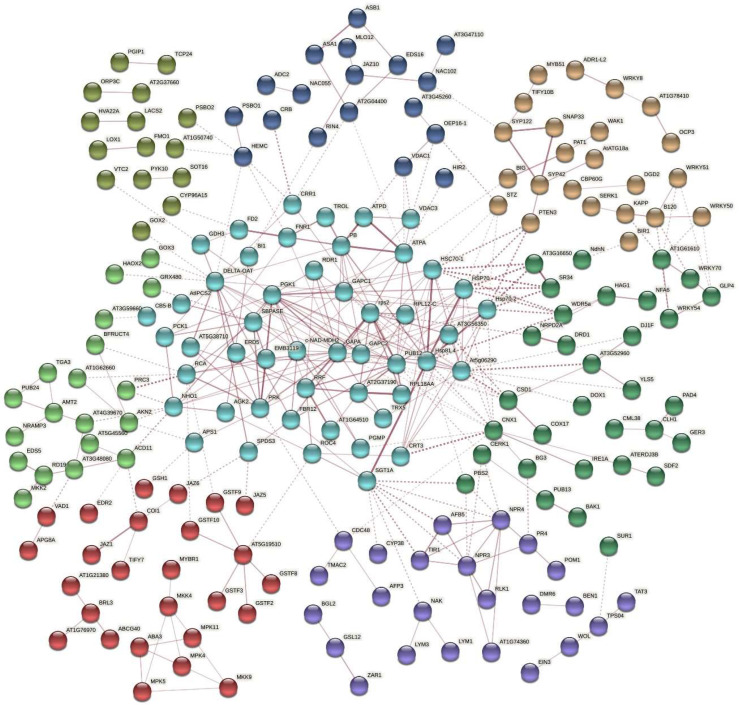
A network of protein–protein interactions based on Arabidopsis homologs of the DEGs. The image was produced on the STRING platform. The proteins are clustered and shown by their nodes in different colors. Thickness of edges indicates different confidence levels. Dotted lines are the edges between different clusters.

**Figure 6:**
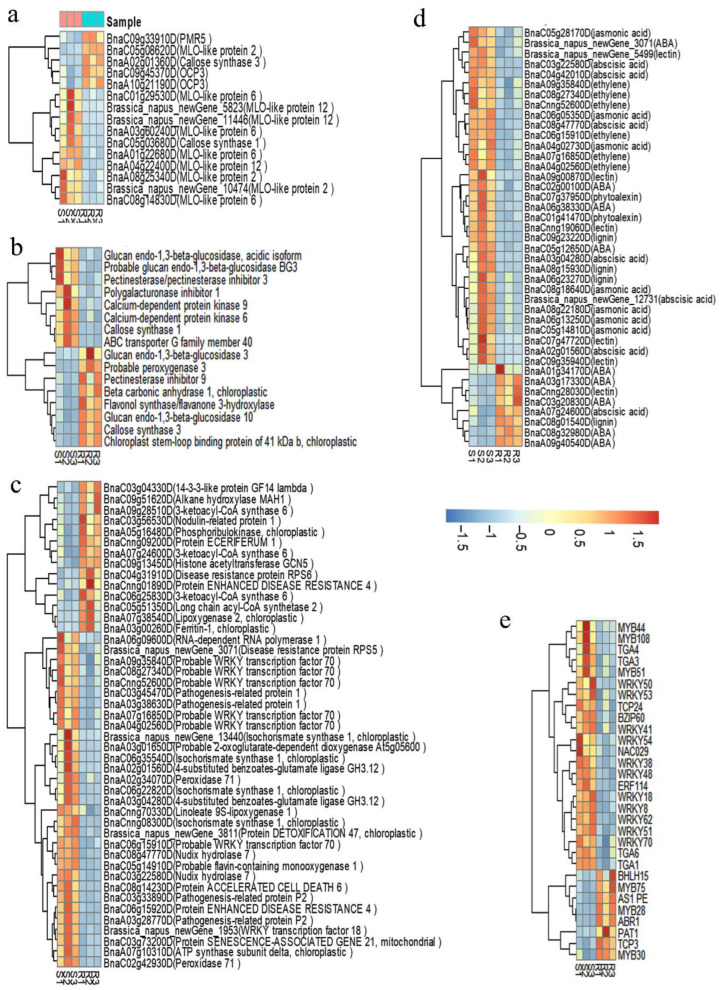
Heatmap of five subsets of the selected differentially expressed genes in the foliar transcriptomes of the S/R plants in the BC_1_F_3_ segregated population. These genes encode the proteins related to (**a**) PM S/R, (**b**) cell wall structure, (**c**) disease resistance, (**d**) transcription factors and (**e**) plant hormone signaling, respectively.

The DEGs related to disease resistance and stress responses were matched through the STRING database to obtain the protein–protein interaction network (Figure 5). Some important proteins clustered in the network including (1) LOX1-LOX2-PAE1-HSP70-PRC3, (2) COI1-JAZ1-JAZ5-JAZ6-JAZ10-TIFY7-TIFY10B-EIN3, (3) WRKY51-WRKY50-WRKY70-WRKY54, (4) BAK1-MEKK1-BIR1-bZIP60-TET8-EXO70B2-XBAT34-MPK11-NUDT7, (5) ATPD-TROL-EMB3119-CRB-PSBO1-PSBO2-GOX2-SBPASE-PRK and (6) RPS2-EMB2270-ATARCA-SAC52-EMB2386-PGY2-RPL27AB-RPL21A-RPL18AA-At1g52300. A heatmap (Figure 6) showed obvious differences in the expression levels of five subsets of interesting DEGs encoding proteins related to (a) S/R to PM, (b) cell wall structure, (c) disease resistance, (d) transcription factors and (e) plant hormone signaling among the S/R plants.

### 2.6. The Expression Level of the Selected Genes Detected by qPCR

**Figure 7:**
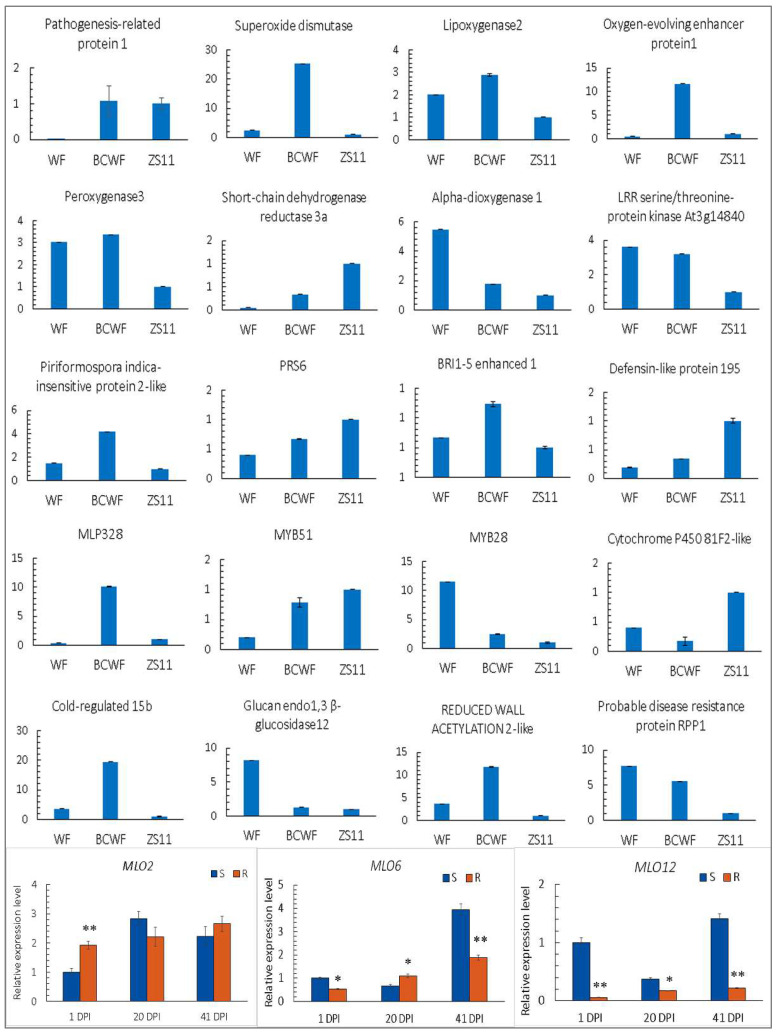
The expression levels of the 20 selected genes detected by qPCR in ‘WF’, ‘BCWF’ and ‘ZS11’ and *BnMLO2*, *BnMLO6* and *BnMLO12* in the S/R plants. Symbols above the bars indicate significant differences at *p* < 0.05 (*) and *p* < 0.01 (**).

The expression levels of 20 DEGs of interest were further compared among the immune ‘WF’, susceptible ‘ZS11’ and highly resistant ‘BCWF’ after PM inoculation via qPCR (Figure 7). These genes represent some vital genes related to defense responses, such as PR-1, SOD, Lox2, OEE1, MYB28, MYB51, Peroxygenase 3, Alpha-dioxygenase 1, LRR kinase At3g14840, MLP-like protein 328, PII-2, Cold-regulated 15b, BEN1, RPP1, Glucan endo-1,3-beta-glucosidase 12, RWA2-like, CYP81F2, SDR3A, Defensin-like 195 and RPS6. The results showed that the expression levels of these genes in ‘WF’ and ‘ZS11’ were largely consistent with the trends in the transcriptomic comparison. Furthermore, the expression of BnMLO2, BnMLO6 and BnMLO12 after PM inoculation is shown in Figure 7. The overall expression level of BnMLO2 was higher than that of BnMLO6 and BnMLO12. The expression levels of BnMLO6 and BnMLO12 in the S plants were higher than those in the R plants in the early and late stages, possibly indicating their vital roles in PM resistance.

## 3. Discussion

Many PTI-related genes, encoding various protein kinases with particular domains, were expressed highly in the R plants, such as *LysM receptor-like kinase* (*LysM-RLK*), LRR receptor kinases *ERECTA, BAK1* and *BAM2* and lipid transfer protein kinases *ERL1* and *ERL2*. These receptor kinases regulate the metabolic activities of mesophyll cells and participate in the recognition of pathogen chitin and peptidoglycan [18,19]. In the S plants, the highly expressed genes encoding protein kinases were mainly receptor protein kinases, which can be assigned to three subsets. The first subset includes MPKs, MKKs and MEKKs. MEKK1, MKK1/MKK2, MKK4/MKK5 and MPK3/MPK6 construct a MAP kinase signaling cascade that modulates the expression of genes in defense by negatively regulating innate immunity [20]. The second subset includes RLK6, CRK45, CRK11 and APK1, which also respond to PAMPs and participate in the stress response and PCD mediated by hormones such as ethylene, SA and ABA. The rest are serine / threonine protein kinases, such as brassinolide receptor protein kinase BRL3 and RLK5, which is involved in ethylene and salicylic acid-dependent cell death.

Our transcriptome analysis shows that there are many ETI-related genes involved in the resistance to PM. These genes mainly include disease resistance-enhancing protein *EDR2* (involved in SA-mediated PM resistance), *EDR4, EDR2L*, RPW8-like protein *HR3*, *PMR5* (connecting pectin and other cell wall polysaccharides), 14-3-3-like *GF14 lambda* (a positive regulator of RPW8 [21]), *RPS4* [22], RPS5 and *RPS6*. The other interesting genes expressed highly in the S plants were TIR-NB-LRR receptor-like *RLM1, DSC1* and *DSC2*, pathogenesis-related *PR-1* (a marker of the plant immune signal of salicylic acid-mediated SAR), receptor *RIN4* (negative regulation in PTI), *ACD6*, *ACD11* (involved in R protein-mediated defense and programmed cell death) and *RPP* (a ULP1-NBS-LRR protein promoting cell death). Plasma membrane-localized multifunctional protein RIN4 plays an important role in both PTI and ETI [23]. Another important gene that is up-regulated is *bZIP22/TGA3*, which interacts with *NPR1, NPR3* and *NPR4* and plays a role in inducing SAR via regulating the expression of *PR-1* [24].

### 3.1. The Possible Contribution of Cuticular Wax and Callose to PM Resistance

The outer-most layer of the plant surface is a hydrophobic cuticle, consisting of cutin and cuticular waxes that protect them from various stresses. The composition of epidermal wax mainly includes ultra-long chain fatty acids, aldehydes, alkanes, secondary alcohols, ketones, primary alcohols, and wax esters [25]. Alkanes can form a smooth wax film without a crystal structure, primary alcohols often form lamellar structures, while most secondary alcohols or diketones form columnar or tubular crystal structures [26]. Our study showed that the leaf wax of PM-resistant plants formed many needle-like or tubular but less flake-like particles. Therefore, the resistant plants may produce more secondary alcohols or diketones, which are conducive to resistance to PM invasion. The elevated expression of several wax biosynthesis genes in the R plants, including *KCS6* coding for ketoyl-COA synthase, *CER* for super long-chain aldehyde decarboxylase and *MAH1* (alkane hydroxylase), may not increase the total wax content but modulate some particular wax components—for example, secondary alcohols and ketones via *MAH1* [27].

It is generally assumed that callose papillae reinforce the cell wall and physically block or hinder the invasion of pathogens [9]. Callose is produced when plants are stimulated by fungal penetration and the deprivation of papillae callose in a *cals12*/*pmr4* mutant enhanced the penetration of *Blumeria graminis* f. sp. *hordei* on Arabidopsis [8,9]. However, callose is not always linked to PM resistance and post-invasive resistance in *pmr4* mutants relies on hyper-induced SA responses [13]. We found that callose accumulation in the resistant rapeseed accessions was lower than in the moderately resistant and susceptible plants because the highly resistant plant can prevent cell penetration through other resistant mechanisms. Consistent with the callose fluorescence density, the *CalS12* expression levels in the mixed samples of the resistant individual plants were higher than those of the susceptible plants in the early and middle stages of inoculation.

### 3.2. Influence of the Composition and Architecture of Plant Cell Wall on PM Infection

A set of genes encoding proteins participating in the morphogenesis and integrity of the cell wall were expressed highly in the R plants—for example, *PMR5, PGIP1, PMEI9, RWA2, PDCB1* and *C/VIF2,* which encode the key proteins in cell wall defense. RWA2 is particularly important for pectin acetylation [28]. PMR5 is involved in the modification of pectin and cell wall polymers and some *pmr5* mutants are resistant to PM species [14]. We also found that the expression of *GDP-L-galactose phosphorylase 1/VTC2* was higher in the R plants but *Mannose-1-phosphate guanylyltransferase 1/CYT1* was higher in the S plants. The two proteins play essential roles in plant growth and the cell wall architecture via the biosynthesis of the antioxidant ascorbate [29]. Furthermore, *WAK1* and *WAKL10* encoding wall-associated receptor kinases were expressed highly in the S plants. The binding of WAK1 and WAKL10 with pectin may control cell expansion, morphogenesis and development [30].

### 3.3. HR and Systemic Acquired Resistance Are Important for the Establishment of Moderate Resistance in Rapeseed Plants

HR, the formation of necrotic lesions at the site of infection, was observed on the moderately resistant and susceptible plants, leading to SAR. SAR is preceded by the accumulation of SA and induction of a set of PR genes. A number of genes related to HR were activated by PM infection, such as *BAK1, PAD4* and *EDS1*. *BAK1* controls the expression of genes associated with innate immunity and controls PCD by negatively regulating the BR-independent cell-death pathway [31]. The Arabidopsis *EDS1* and *PAD4* encode lipase-like proteins that function in R gene-mediated and basal plant disease resistance [32]. PAD4 is required for PCD triggered by NBS-LRR resistance proteins (e.g., RPS4 and RPW8) and is necessary for the SA-dependent SAR response but, together with EDS1, it seems to repress the ethylene/jasmonic acid (ET/JA) defense pathway [33]. Growth factor gene *NUDT7* is a negative regulator of basal immunity via negative control of EDS1 signaling [34]. *NIM1* (Non-inducible Immunity 1)/NPR1 (Non-expresser of PR genes 1) is a key positive regulator of SAR and PR gene activity is regulated at the level of the redox-dependent nuclear localization of NPR1 [35]. We found that *Phosphatidylinositol:ceramide inositolphosphotransferase 2* (*IPCS2*), termed Enhancing RPW8-Mediated HR-Like Cell Death 1, which plays an important role in regulating defense-related plant programmed cell death (PCD) [36], was expressed highly in the R plants. Loss of function in *ACO4* or *EIN3* increased RPW8.1-mediated cell death and the overexpression of EIN3 significantly compromised cell death in Arabidopsis [37]. In our study, the expression of *ACO4* and *EIN3* homologs was elevated in the S plants. This may be a response to compromise cell death. In addition, the genes coding for dehydration response proteins, such as cysteine proteases RD19A, R19B, RD21A and RD21B, which play roles in plant immunity and act as elicitors to stimulate PCD [38], and Hypersensitive Response 2 (HSR2)/mitochondrial outer membrane protein VDAC3 were expressed highly in the S plants. Other highly expressed genes in the S plants were *PR-1* (a marker of SA-mediated SAR), *ACD6* (*Accelerated Cell Death 6*), *ACD11, ACA11* (negatively regulates PCD), *ATG8a* and *RPP* (promotes cell death).

ETI triggers the immune system to activate plant HR, which is often accompanied by the outbreak of ROS. Respiratory burst oxidase homolog protein F (RBOHF) generates ROS during incompatible interactions with pathogens and is important in the regulation of HR. It was found that in the R plants such genes as *DOX1* (producing lipid-derived molecules to regulate the protection against oxidative stress), *GLO3* (glycolate oxidase regulating ROS-mediated signal transduction) and *chloroplastic peroxiredoxin-2E* (*PRXIIE*) were highly expressed. DOX1 is a pathogen-induced oxygenase that catalyzes the primary oxygenation of fatty acids into oxylipins, mediates protection against oxidative stress and promotes local and systemic plant defense in an SA-dependent manner, including the establishment of SAR in response to incompatible interactions [39]. In the S plants, *GH3.12*, encoding a 4-substituted benzoate-glutamate ligase, was required for the establishment of HR upon incompatible interaction and subsequent SAR. Moreover, *EDR4* and *EDR1* are also important genes for H_2_O_2_ accumulation, callose deposition and HR-like lesions at the infection site [40].

## 4. Materials and Methods

### 4.1. Plant Materials

The resistance gene donor of Ethiopian mustard strain ‘WF’, susceptible cultivar ‘ZS11’ of *B. napus*, their F_1_ hybrid backcrossed to ‘WF’ (BCWF, highly resistant) and two BC_1_F_3_ populations ‘W7-6’ and ‘W8-3’, which were derived from the progeny of the F_1_ backcrossed to ‘ZS11’ [2], were used in this study. R and S plants segregated from the BC_1_F_3_ populations were mixed as two bulks in the comparison of traits related to PM resistance. The plant accessions were grown in a greenhouse at a maximum/minimum temperature of 25 °C/15 °C under a photoperiod of 12 h/12 h. The seedlings at the five-leaf stage were well moisturized by a mist sprayer and spontaneously infected by placing diseased plants nearby to mimic the natural inoculation of *E. cruciferarum*. The diseased plants were grown in ten pods with unfixed positions and the *E. cruciferarum* spores produced on their leaves were placed near fans to ensure even dispersion throughout the whole greenhouse. Observed by naked eyes, the colonies of mycelia were expected to be visible on the fully expanded leaves at 10 to 15 DPI, and PM infection was expected to reach the peak at 20 to 30 DPI. The process was expected to last over two months before the first infected leaf’s senesce. Thus, the upmost fully expanded leaves at 1, 20 and 41 DPI were used in the following experiments. The process of PM pathogenesis and the criteria for the evaluation of the PM resistance levels were described in our previous study [2].

### 4.2. Microscopic Observation

The upmost fully expanded leaves in the plants with different levels of disease incidence were cut into pieces, and fixed in Carnoy’s solution I (glacial acetic acid:ethanol = 1:3). Tissue samples were then infiltrated by liquid paraffin and embedded in paraffin wax. The wax blocks containing leaf samples were cut into slices using a Leica ASP200S microtome (Leica Microsystems, Wetzlar, Germany). The obtained slices were stained with 0.5% safranin-O, followed by 0.1% fast green and observed under a light microscope. Different leaf segments collected from 5 plants for each plant accession were observed.

To observe the process of PM infection and cuticular wax on the leaf epidermis, the leaves in the same position of the plant accessions were sampled. They were air-dried at 40 ˚C, gold-coated and observed under a Hitachi S4800 scanning electron microscope (Hitachi High-Tech, Tokyo, Japan).

### 4.3. Assay of The Leaf Cuticular Wax

We utilized a gravimetric method to roughly quantify the leaf wax. For this purpose, the upmost fully expanded leaves at the different stages (1, 20 and 41 DPI) were collected and the surface dust and mycelia were wiped off. The leaves were cut into pieces of the same size and each piece was washed in 50 mL of chloroform for 30 s in a glass cup. Then, the leaf was taken out and chloroform was evaporated at 40 °C. The weight of wax, the residual in the cup, was estimated as the difference between the beginning and final weights of the cup.

### 4.4. Determination of Callose Content in Leaves

The upmost fully expanded leaf samples of S/R plants in the BC_1_F_3_ population at 1, 20 and 41 DPI were stained with a solution of 1% Aniline Blue and visualized under a fluorescence microscope. The average optical density of callose deposition was quantified in the software ImageJ (2 Mar 2020, v2.0.0, https://imagej.nih.gov/ij/).

### 4.5. Real-Time Quantitative PCR (qPCR)

The total RNA in the leaves of the S/R plants challenged by PM was extracted using an RNA Extraction Kit (TaKaRa, Shiga, Japan) and reverse-transcribed into cDNA using the FastKing RT Kit (Tiangen, Beijing, China). qPCR was performed on a QuantStudio 7 Real-Time PCR System (ThermoFisher Scientific, MA, USA) using SYBR Green dye. The PCR primers for rapeseed *CALS12*, *MLO2*, *MLO6*, *MLO12* and 20 other selected genes (Table 1) were designed by Primer-BLAST (5 May 2020, v4.1.0, https://www.ncbi.nlm.nih.gov/tools/primer-blast). Each gene was tested in three replicates and rapeseed *actin7* served as the internal reference gene for data normalization.

### 4.6. Transcriptomic Comparison between the Susceptible and Resistant Plants

The leaf samples were taken from the identified S/R seedlings in the BC_1_F_3_ population during the peak period (20 DPI) of PM infection. The upmost fully expanded leaves of susceptible and resistant plants were carefully collected after the removal of the surface dust and mycelium. Total RNA was extracted using Trizol reagent and subjected to quality control with the Agilent Bioanalyser 2100 (Agilent Technologies, Santa Clara, CA, USA). The cDNA libraries of the S/R plants, with three biological replicates, were prepared using a standard Illumina protocol and were sequenced on an Illumina Novaseq6000 platform (Illumina Inc., San Diego, CA, USA). All the processed sequences (deposited in NCBI’s Gene Expression Omnibus with the accession number GSE188377) were mapped onto the reference genome sequences of *B. napus* cultivar ‘Darmor-bzh’ (10 April 2020, v5, https://www.genoscope.cns.fr/brassicanapus) using the alignment software HISAT2 (3 January 2020, v2.2.0, https://cloud.biohpc.swmed.edu/index.php/s/hisat2-220-source). Through calculation and standardization of RPKM (reads per kilobase of exon model per million mapped reads), a comparison between two sample groups was performed using DEseq2 (3 January 2020, v1.36.0, https://bioconductor.org/packages/DESeq2/), using Log_2_(Fold change) ≥ 1 and False Discovery Rate < 0.01 as the criteria to identify DEGs. Gene Ontology (GO) and KEGG pathway enrichment analysis of the DEGs was implemented using the GOseq R packages (3 January 2020, v1.48.0, https://bioconductor.org/packages/goseq/) and KOBAS (8 May 2021, v3.0, http://kobas.cbi.pku.edu.cn/kobas3/) software. The DEGs were searched in the Arabidopsis genome in the STRING database (15 May 2021, http://string-db.org/) to obtain the predicted protein–protein interaction of these DEGs. The possible functions of the DEGs and corresponding proteins were identified in the UniProtKB database (15 May 2021, https://www.uniprot.org/).

## 5. Conclusions

The comparative transcriptome analysis in this study identified several important pathways and a set of pivotal genes involved in PM resistance, which are multi-layered at both the pre- and post-penetration stages. For the resistant plant accessions used in the present study, the expression of immune-related genes accompanying the early event of penetration resistance can strengthen disease defense. For the susceptible plants, however, the initial defense is insufficient, and the hyphae invasion led to the elevated expression of a wider range of genes in later defense responses. Therefore, the initial resistance to cell penetration may be of great significance for the overall disease resistance of rapeseed to PM. The findings shed new light on the phenotypic characterization and transcriptional regulation of brassica PM resistance.

## Figures and Tables

**Table 1:** The primers designed for qPCR test of the selected genes.

Gene	Annotation	Primers Pair (5′→3′)
*ACTIN7*	Beta Actin 7	CATCCCTCAGCACCTTCC CCAACCTTAGCACTTCTCC
*CalS12*	Callose synthase 12	GTGGCATCAGTTCTTTGGCG AGCTTCAATCTATGAATGGCGTC
*MLO2*	Mildew Resistance Locus 2	AAGAAGCACAAGCAGGCTCT TGCAAGGGTGCATTGTTGAC
*MLO6*	Mildew Resistance Locus 6	CTTCTGGTTCGGTCGTCCAC TTAGACCGAATTCATACGTACTCC
*MLO12*	Mildew Resistance Locus 12	GAACCGGGCGATGATCTCTT GCCAGTTGAAATGCATTCGTG
*BnaC03g45470D*	PR1, pathogenesis-related protein 1	TCACAACCAAGCACGACAGG GTTCTCTCCATAAGGCCCACC
*BnaC08g42970D*	Superoxide dismutase [Cu-Zn]	CACCCATGAAGGAAACGGTG CGATTAGCATCCTCCGGTGC
*BnaA02g11430D*	Lox2, lipoxygenase 2, chloroplastic-like	CCGTCTGTGAACAAAGTGAGAG ACAATGAGTCCTCAGCCAGT
*BnaC07g46350D*	OEE1	TCACCGTCAAAGCAGAAGGT CTGTCACTGCAGCGTAATCT
*BnaA03g40190D*	MYB28-like	CAATGCCTTCCCTGTCTCGT CCAATCTGCTCAGAGAAGCCA
*BnaA05g10200D*	Probable peroxygenase 3	GTACGTAGCGACTTGGAGGAG GGGTAGACAATACCGTCGCC
*BnaA05g24690D*	Probable LRR-RLK At3g14840	GGTCCAATTCCTCCCGAATG TGAAATCAAGGCCAAGGAACT
*BnaA01g31910D*	MLP-like 328	ACATCTTCCCTGACGCCATC ACGTGACCCTCAAGTCCTCT
*BnaA07g27410D*	PII-2, Piriformospora indica-insensitive protein 2-like	ATGGAGAAAACAGAGAAAGCTGC AGATCACAAGTTACACCCTGGA
*BnaA03g56740D*	Cold-regulated 15b	ATTCTTCTTTCCCCAGCGGC CGCGTAATCCGAAGCTCTCT
*BnaC04g53100D*	BEN1, BRI1-5 ENHANCED 1-like	CCCAGTTTCAGCTACCTTCAGT ATTTTTAACCCCCAACATAAAGAGT
*BnaC06g11870D*	Probable disease resistance protein RPP1	CGCCAACAAGAGATTTCGAGG ACATATTTCGCCGTGGGAGA
*BnaCnng77750D*	Alpha-dioxygenase 1	TGTGACGCACTTTGAATGACT CACGAAAGCAAAGCAAATAGCA
*BnaA05g30820D*	REDUCED WALL ACETYLATION 2-like	CACACTTCACTGTTCGTGTAACT ACGAAGAACCAAAGCGCGATA
*BnaA03g11520D*	Glucan endo-1,3-beta-glucosidase 12	CCGTGTTCGCGGCCAT GACGTTAAGCGGCTCATTCG
*BnaC02g11750D*	Cytochrome P450 81F2-like	TATAGAATCAAACCCACCCACC CGATGGGAAACGGAGTTGGT
*BnaC03g72480D*	Short-chain dehydrogenase reductase 3a LOC106438316	TGTCGGGAAGCAGACTAGATG GGAAAGAGCAACGCTTAGACC
*BnaC04g52990D*	Defensin-like protein 195 (loc106394639)	GAGGGAATACGGTGGCGAT TAGTCGCATAAGCACCTGAC
*BnaA09g44500D*	Transcription factor MYB51	TACCTCCTACGTTAACCGTCAC AGAGAGATGATTGAAAGTTCCTCGT
*BnaC04g12970D*	Disease resistance protein RPS6-like	CGTATGGCTGGACAGGTTGA GAGGCACCGTTCAGCTAGAG

## Data Availability

All the data generated or analyzed during this study are included in this published article and its supplementary information files. The RNA-seq data supporting the results of this article are available at NCBI’s Gene Expression Omnibus under accession number GSE188377.

## Appendix A

**Dataset A1**. RPKM values and annotation of the differentially expressed genes involved in defense responses (The file was attached in the package containing the manuscript).
